# Minimal residual disease detection in lymphoma: methods, procedures and clinical significance

**DOI:** 10.3389/fimmu.2024.1430070

**Published:** 2024-08-12

**Authors:** Sijun Zhang, Xiangyu Wang, Zhenzhen Yang, Mengjie Ding, Mingzhi Zhang, Ken H. Young, Xudong Zhang

**Affiliations:** ^1^ Department of Oncology, The First Affiliated Hospital of Zhengzhou University, Lymphoma Diagnosis and Treatment Center of Henan Province, Zhengzhou, Henan, China; ^2^ School of Public Health, Zhengzhou University, Zhengzhou, Henan, China; ^3^ Division of Hematopathology, Duke University Medicine Center, Duke Cancer Institute, Durham, NC, United States

**Keywords:** lymphoma, minimal residual disease, liquid biopsy, circulating tumor DNA, minimal residual disease monitoring

## Abstract

Lymphoma is a highly heterogeneous lymphohematopoietic tumor. As our understanding of the biological and pathological characteristics of lymphoma improves, we are identifying an increasing number of lymphoma subtypes. Genotyping has enhanced our ability to diagnose, treat, and monitor the prognosis of lymphoma. Despite significant improvements in treatment effectiveness, traditional methods for assessing disease response and monitoring prognosis are imperfect, and there is no significant improvement in overall remission rates for lymphoma patients. Minimal Residual Disease (MRD) is often indicative of refractory disease or early relapse. For lymphoma patients, personalized MRD monitoring techniques offer an efficient means to estimate disease remission levels, predict early relapse risk, and assess the effectiveness of new drug regimens. In this review, we delve into the MRD procedures in lymphoma, including sample selection and requirements, detection methods and their limitations and advantages, result interpretation. Besides, we also introduce the clinical applications of MRD detection in lymphoma.

## Background

Lymphoma is a type of malignancy resulting from monoclonal proliferation that arises either from lymph nodes or extranodal lymphoid tissue. Despite considerable progress in therapeutic strategies such as chemotherapy, targeted therapy, and immunotherapy, curative outcomes remain elusive due to the persistence of minimal residual disease (MRD). MRD is a diminutive population of malignant cells that continue to survive post-therapy, posing a significant hurdle to achieving a cure. MRD may persist in a stable state for an extended period or gradually be eradicated by the body’s immune system. However, these cells can provoke molecular relapse in patients, even those who have achieved clinical remission. Recent research underscores the significance of MRD detection in the peripheral blood (PB) or bone marrow (BM), demonstrating its superior prognostic potential in predicting patient survival outcomes, even beyond conventional predictive factors (1–7). The use of MRD in directing personalized treatment for lymphoma patients is currently being explored, with prospects for its application across a broader spectrum of lymphoma subtypes in the future. MRD has the potential to become a pivotal tool in lymphoma management, contributing to improved patient outcomes and enhanced overall treatment efficacy. However, residual tumor cells that are not fully detectable can lead to inaccurate MRD assessment if inappropriate detection methods with lower sensitivity are used. Therefore, it is important to choose highly sensitive and specific techniques for detecting MRD in different lymphoma subtypes to ensure accurate identification. Through personalized MRD detection for various lymphoma subtypes, it is possible to predict prognosis pre-treatment, stratify risk, dynamically monitor treatment effectiveness, adjust treatment regimens, and track molecular relapse during periods of clinical remission.

**Figure 1:**
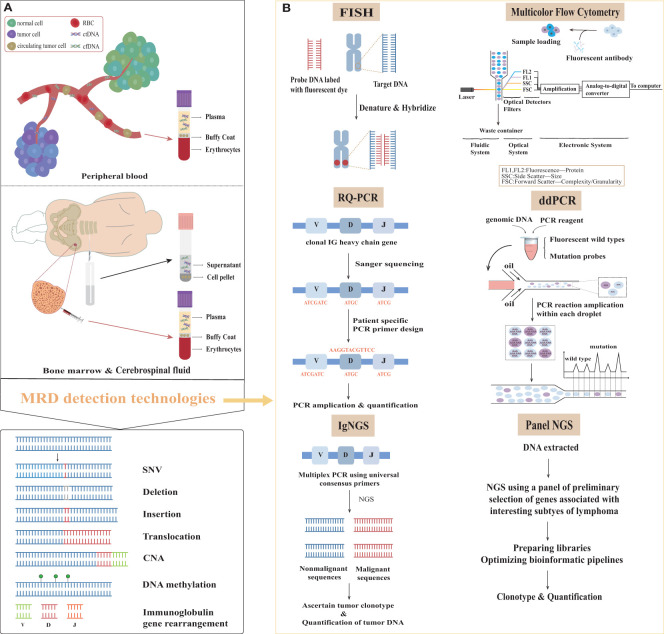
Samples and methods for minimal residual disease detection in lymphoma. **(A)** The primary sources of samples and overview of molecular variants of interest in the surveillance of lymphomas. **(B)** Minimal residual disease assessment methods in patients with lymphoma. MRD, minimal residual disease; ctDNA, circulating tumor DNA; cfDNA, circulating free DNA; SNV, single nucleotide variation; CNV, copy number variation; V, variable; D, diversity; J, joining; IG, immunoglobulin; RQ-PCR, real-time quantitative polymerase chain reaction; ddPCR, digital droplet polymerase chain reaction; IgNGS, immunoglobulin next generation sequencing; Panel NGS, panel-directed next generation sequencing; FISH, fluorescent *in situ* hybridization.

In recent decades, liquid biopsy’s potential in detecting lymphoma MRD has gained significant attention among scholars (**Figure 1**). Liquid biopsy involves using various molecular detection techniques to analyze tumor-specific genetic information found in bodily fluids like PB, cerebrospinal fluid (CSF), and urine. This method primarily focuses on circulating tumor cells (CTCs), circulating free DNA (cfDNA), circulating tumor DNA (ctDNA), tumor-educated platelets (TEPs), and exosomes. cfDNA refers to all DNA released from cells—whether they’re healthy, inflamed, or cancerous—following processes like apoptosis or necrosis. In contrast, ctDNA specifically relates to the portion of cfDNA derived from tumor cells, reflecting the dynamic genetic changes in malignant cells, such as mutations, methylation, amplification, or rearrangement. Notably, while ctDNA is a small subset of cfDNA, its detection is vital for an accurate MRD assessment. Such data plays a pivotal role in quantitative detection methods (8, 9). (**Figure 1A**) Lymphomas exhibit significant heterogeneity, often presenting multiple lymphoma foci in different anatomical locations within a single patient. Circulating tumor DNA-based liquid biopsy offers a stark contrast to traditional tissue biopsy techniques. Being minimally invasive, it detects cell-free DNA from diverse tissue sites. This provides a broader perspective on gene mutations for diagnostics, identifies mutation patterns linked to adverse prognoses, and enables dynamic monitoring of MRD alterations during treatment. By quantitatively analyzing ctDNA, clinicians can obtain accurate information on MRD levels, which aids in informed disease management and treatment strategy formulation (10).

In most lymphoma cases, cfDNA offers a more dependable approach to MRD detection than CTCs. Among the diverse liquid biopsy types for lymphoma, peripheral blood stands out as the most extensively researched. It consistently shows detectable ctDNA levels, even in the early disease stages. Notably, plasma, compared to serum, exhibits fewer contaminations from germline DNA, making it the favored medium for ctDNA genotyping (9). In Primary central nervous system lymphoma (PCNSL), ctDNA detection is most successful in CSF. However, when CSF collection is difficult or risky for the patient, plasma ctDNA analysis becomes a valuable alternative. Mutter et al. substantiated this assertion by examining CSF and plasma samples from newly diagnosed PCNSL patients before treatment. They found ctDNA in 78% of plasma samples and 100% of CSF samples (11). Numerous studies have delved into the clinical relevance of ctDNA across various lymphoma subtypes, including Diffuse large B-cell lymphoma (DLBCL) (12), MCL (2), Hodgkin lymphoma (HL) (13), Follicular lymphoma (FL) (10), PCNSL, Natural-killer/T-cell lymphoma (NKTCL) (14), and Peripheral T cell lymphomas (PTCL) (15). Targeted sequencing of paired plasma and tumor tissue samples taken concurrently has shown that they largely share a consistent somatic mutation profile (15, 16). Beyond somatic mutations, ctDNA in plasma also carries additional genetic insights, including chromosomal rearrangements, methylation patterns, and copy number variations. For instance, Cai et al. (14) studied the methylation spectrum of Extranodal natural killer/T cell lymphoma (ENKTL) plasma samples, devising both diagnostic and prognostic prediction models rooted in ctDNA methylation markers.

This review primarily focuses on MRD procedures in lymphoma, including sample selection and requirements, currently available MRD detection methods (**Figure 1B**) and their selection, their limitations and advantages, result expression, and their potential clinical applications.

## The selection and requirement of samples

### The selection of samples

Both PB and BM are appropriate options for sample selection in MRD detection. In a study focusing on PTCL with bone marrow infiltration, paired BM and PB samples were collected. The detection of MRD was conducted through FCM, and the results revealed that the sensitivity of PB samples was 85.71%, with a specificity of 100% compared to BM samples. This supports that the PB samples is feasible in clinical (17). The PB MRD negativity rate was higher than BM during the end of treatment. This difference could be attributed to either the faster clearance rate of the disease in PB compared to BM or the lower sensitivity of PB in detecting MRD. Regardless, both types of samples offer valuable insights. It is noteworthy that during the initial stages of treatment, an MRD-negative result in either a PB or BM sample is associated with longer PFS (3). For central nervous system lymphoma (CNSL), cfDNA concentrations were particularly low in CSF as compared to plasma samples. In contrast, ctDNA levels were increased in CSF as compared to plasma. This suggests that CSF ctDNA may possess a superior capacity for MRD detection compared to plasma ctDNA (18, 19). Nevertheless, plasma ctDNA can also serve as a prognostic pool for patients with CNSL, particularly during post-treatment assessments. Plasma ctDNA offers insights into the gene mutation landscape associated with CNSL. The rate of plasma ctDNA mutations concordant with those in tumor tissue DNA was 56.7% (97/171), while the concordance rate between CSF ctDNA and tumor tissue DNA was 74.0% (94/127) (18).

The choice of sample for MRD detection depends on the lymphoma subtype and specific purpose. Bone marrow resource samples are not recommended when the patient does not have bone marrow invasion or has achieved bone marrow remission following treatment. CSF seems to be a more favorable choice for individuals diagnosed with CNSL, encompassing both primary central nervous system lymphoma (PCNSL) and secondary central nervous system lymphoma (SCNSL). However, When the objective of the study is to track disease recurrence in the follow-up period, BM or CSF samples are no longer ideal for patients who have achieved complete remission (CR) or bone marrow remission. Both bone puncture and lumbar puncture remain invasive procedures. PB samples are a more accessible alternative, potentially alleviating the significant burden of clinical sampling and enhancing patient adherence.

### The requirements of samples

The quality of samples exerts a significant influence on the results of clinical studies related to MRD detection. Factors such as the sampling method, preservation technique, and transportation of samples can all affect the detection rate of abnormal clonotypes in the samples (20). Therefore, enhancing the quality of these samples is crucial for ensuring the accuracy and reliability of MRD assessment.

The levels of peripheral blood cfDNA in lymphoma patients are typically higher than those in healthy individuals. However, an increase in cfDNA content is not exclusive to lymphoma or other malignant tumors. Various physiological conditions, such as exercise, inflammation, tissue damage (including trauma or infarction), and pregnancy, may lead to elevated cfDNA levels. For lymphoma patients, the occurrence of such complications may reduce the ctDNA percentage in peripheral blood. In the quest for accurate ctDNA detection, continuous improvement in the detection limit of available technologies is necessary (9, 21). To ensure precise outcomes, it is vital to avoid the lysis of peripheral blood mononuclear cell (PBMC) and prevent genomic DNA contamination. Implementing standard procedures for sampling and transportation is paramount to obtaining accurate results (8, 9). Lymphoma patients exhibit considerable variability in cfDNA concentration, with plasma cfDNA levels ranging from approximately 6.5 ng/mL in FL to around 650 ng/mL in primary mediastinal B-cell lymphoma (PMBCL). These cfDNA levels are significantly correlated with the tumor stage. A minimum of 10 milliliters of blood (equivalent to approximately 4-6 milliliters of plasma) is typically required to obtain enough cfDNA molecules for subsequent analysis (21). When collecting peripheral blood with an EDTA tube, it is crucial to separate the plasma from the blood within 6 hours after collection to prevent cell DNA contamination from PBMCs (9, 21). Utilizing tubes containing cell stabilizers can minimize PBMC contamination and maintain stability for approximately 7 days at room temperature (8, 9, 21). For cerebrospinal fluid samples, a minimum of 1-3 ml of cerebrospinal fluid should be collected. The sample should be centrifuged within 2 hours if a cerebrospinal fluid sampling tube is used. The supernatant should be taken and stored at -80°C (18, 19).

Additionally, the presence of clonal hematopoiesis of indeterminate potential (CHIP) and germline variants can create confusion, obstructing the differentiation of ctDNA from the cfDNA background released by normal cells (9, 21). To counteract the confounding effects of CHIP, whether PB or CSF, it is advised to collect the lower cell pellet for sequencing analysis of cfDNA and derived DNA related to hematopoietic stem cells, thereby eliminating background noise and improving the accuracy of the results (19).

## MRD detection methods and status

CT/MRI and FDG-PET/CT are important evaluation tools in the Lugano evaluation criteria. By utilizing Fluorine-18 2-fluoro-2-deoxy-D-glucose (^18^F-FDG), FDG-PET/CT provides a comprehensive view of both the anatomical and functional characteristics of lymphomas compared to CT/MRI. FDG-PET/CT facilitates the quantitative assessment of tumor volume, using markers of metabolically active tissue such as Metabolic Tumor Volume (MTV) and Total Lesion Glycolysis (TLG) (22). For lymphoma subtypes that do not exhibit high uptake of FDG, such as chronic lymphocytic leukemia/small lymphocytic lymphoma (CLL/SLL), marginal zone lymphoma (MZL) outside the lymph nodes, and certain cutaneous/intestinal T-cell lymphomas (CTCL/EATL), CT/MRI is currently the predominant imaging modality for staging, evaluating tumor burden, and assessing treatment response. Conversely, PET/CT remains the preferred tool for FDG-avid lymphoma subtypes such as HL, DLBCL. Deep learning-based computer-aided diagnosis systems, including Convolutional networks for biomedical image segmentation (U-Net), Cruciform structure guided and boundary-optimized lymphoma segmentation network (CGBO-Net), DL-Convolutional Neural Networks (DL-CNN), and Anatomical-Metabolic Consistency Generative Adversarial Network (AMC-GAN), has the potential to enhance the precision of PET in lymphoma identification and segmentation (23–25). Yet, these imaging modalities are not without drawbacks. They are expensive and come with inevitable radiation exposure concerns, potentially increasing the risk of secondary malignancies. They also exhibit limited sensitivity and specificity, leading to possible false results which can impact clinical decisions or even lead to relapse post CR achievement. These methods also fall short in detecting the MRD level in lymphoma patients who have achieved clinical CR (26, 27).

### Fluorescent *in situ* hybridization

Fluorescent *in situ* hybridization (FISH) can identify copy number variations and gene fusions caused by chromosomal translocations or rearrangements. FISH is a commonly used molecular detection method in clinics. It has proven valuable in various lymphoma subtypes including ALCL, BL, DLBCL, FL, and MCL (28, 29). FISH can detect the most important genetic aberrations at diagnosis. For example, FISH is adept at pinpointing High-grade/large B-cell lymphoma with 11q aberration and identifying rearrangements in MYC, BCL2, and/or BCL6 (30–33). FISH has the capability to identify genetic mutations associated with unfavorable prognosis in lymphoma patients, aiding in risk stratification, such as the TP53 gene (34, 35). Beyond diagnostic applications, FISH is also used to trace clone evolution (4). Recently, FISH has also seen new technological advancements, such as multicolor fluorescence *in situ* hybridization (mFISH), which can detect multiple gene regions on chromosomes at the same time (28). Nevertheless, FISH is restricted to the analysis of predetermined genes and exhibits a relatively low detection efficiency. The absence of predetermined gene mutation sites, which are unknown mutations or rare variants outside the detection range of the kit, may lead to false negative results.

### Multicolor flow cytometry

Multicolor flow cytometry (MFCM) is a technique that differentiates and categorizes cell populations based on their specific cell phenotypes. This is achieved by analyzing light signals emitted from cells that have been tagged with multiple fluorophore-conjugated antibodies. These signals are then processed by electronic systems for interpretation. Flow cytometry for MRD detection eliminates the need for patient-specific polymerase chain reaction primers and exhibits a vast range of applicability (3).MFCM is recognized as an effective method for identifying MRD levels in leukemia (3, 26). 4-color flow cytometry is less sensitive compared to conventional PCR in MCL (36). Remarkably, even the more advanced 8-color flow cytometry doesn’t exceed a detection threshold of 10^-4^. With a cutoff set at 0.01%, flow cytometry achieves an 80% true positive rate compared to Real-time quantitative polymerase chain reaction (RQ-PCR), and it also has a 92% true negative rate (37). Certain lymphomas, such as MCL, FL, MZL, Small lymphocytic lymphoma (SLL), and Burkitt lymphoma (BL), have residual tumor components in the peripheral blood, like circulating tumor cells (CTCs). Flow cytometry can be used to detect these CTCs for MRD detection. Conversely, other lymphoma subtypes like DLBCL and classical Hodgkin’s lymphoma (cHL) typically do not present with CTCs, making them unsuitable candidates for MRD detection using flow cytometry. It’s noteworthy that even for MCL, the subtype most commonly associated with CTCs, MFCM has a reduced sensitivity for MRD detection when juxtaposed with alternative techniques such as RQ-PCR (8, 26). Background interference from normal cells can result in false positive results. The limited quantity of tumor cells for MRD detection, and the potential alteration in immunophenotype on the surface of tumor cells, may lead to false negative results. In addition, the extensive data it generates often demands robust computing power for processing. Subjective interpretations of Flow cytometry results, coupled with varying judgments across observers, stand as notable limitations (38).

### Polymerase chain reaction

In patients with B-cell or T-cell lymphomas, there’s a malignant proliferation of specific B or T cells. As a result, identical immunoglobulin or TCR gene rearranged sequences emerge within the patient’s body. These sequences act as unique molecular markers for lymphomas and are pivotal in monitoring MRD (39, 40).

The detection threshold of real-time quantitative polymerase chain reaction (RQ-PCR) is 10^-5^ (39, 41, 42). RQ-PCR has been employed to analyze diverse subtypes of lymphoma, such as MCL, DLBCL, ALCL, shedding light on the importance of MRD status (43–46). However, RQ-PCR isn’t without its challenges. Although PCR-based methods are more sensitive than MFC, there are still no available molecular markers for certain lymphomas using RQ-PCR (47). Using universal primers, RQ-PCR amplifies genes in the immunoglobulin target region. Subsequently, these amplified sequences are sequenced to accurately design patient-specific primers. The intricate and time-consuming primer design process, compounded by the absence of standardized protocols, impedes the broader integration of RQ-PCR into routine clinical settings (48). The limited quantity of tumor cells for MRD detection, and the low nucleic acid concentration in the specimen, may lead to false negative results.

Digital droplet PCR (ddPCR), when combined with high-throughput sequencing technology, offers sensitive quantitative detection of tumor-specific somatic mutations. It’s been effectively employed for MRD monitoring in MM, MCL, DLBCL and FL (42). A study on DLBCL demonstrated that blood samples could detect potential treatment-related somatic mutations with a sensitivity as high as 0.05%. However, when pitted against panel-NGS, ddPCR’s capability to simultaneously evaluate multiple mutations is limited. It only provides data on previously identified mutations. As a result, ddPCR falls short when it comes to monitoring tumor clonal evolution for MRD detection (26). Contrasting with RQ-PCR, ddPCR stands out as an absolute quantitative PCR method. It’s independent of relative quantitative standard curves and boasts superior sensitivity, particularly at lower levels, which ranges between 10^-4^ to 10^-5^. Another noteworthy advantage of ddPCR is its capability to ensure consistent results across different laboratories, making it a reliable choice (41, 49). The contamination of cfDNA with leukocyte genomic DNA can result in false positive results. The low ctDNA concentration in the specimen, and lack of predetermined gene mutation sites, which are unknown mutations or rare variants outside the detection range of the kit, may lead to false negative results.

### Next-generation sequencing

We need to use more sensitive methods to enable mutation detection of low cfDNA abundance, especially at low Tumor Fraction (TF). Next-generation sequencing (NGS) has become a pivotal method for MRD detection, surpassing the traditional methods of PCR and FCM in terms of sensitivity. While RQ-PCR and MFCM have sensitivities of 10^-5^ and 10^-4^-10^-5^ respectively, NGS boasts a detection limit of 10^-6^, indicating potential advantages for future applications. Taking DLBCL as an example in aggressive lymphomas, the rate of somatic mutation detection varies from 63% to 87% in cfDNA (50–52). In majority of cases, the coincident mutations with ≥69% are observed both in the paired cfDNA and gDNA. In the remaining cases, the number of mutations identified in cfDNA was lower than that observed in the paired gDNA. Of note, most cases in which mutations could not be detected in the cfDNA corresponded to localized disease stages (50). The sensitivity of cfDNA to detect the mutations present in paired gDNA varies from 68% to 97.1%. cfDNA sequencing showed the highest sensitivity when taking into account only the mutations present with ≥20% allelic frequency in gDNA, demonstrating that cfDNA can accurately mirror the profiles of the most abundant clones found in gDNA (50, 52). Taking FL as an example in indolent lymphomas, Fernández-Miranda et al. (10) have evaluated paired cfDNA and gDNA using Panel-NGS. At least one somatic mutation was detected in all tumor gDNA and 74% (17/23) plasma cfDNA. 73% of mutations detected in tissue gDNA were identified in cfDNA. Biopsy-confirmed gDNA mutations are detectable with 84.84% sensitivity in cfDNA when considering the mutations present with ≥18% allelic frequency in gDNA.

Two primary types of NGS-based lymphoma MRD detection exist: the IgNGS, which identifies immunoglobulin or T cell receptor genes through methods like Clono-SEQ, and the panel-NGS, which is a highly-sensitive and panel-directed approach identifying somatic genetic changes through methods such as Cancer personalized profiling by deep sequencing (CAPP-SEQ) and Phased variant enrichment and detection sequencing (PhasED-SEQ) (26, 53). Notably, NGS surpasses RQ-PCR by overcoming its inherent limitations. Unlike RQ-PCR, NGS doesn’t require the design of patient-specific primers and offers enhanced specificity (39). Furthermore, targeted therapies might induce the downregulation or absence of crucial cell surface markers during tumor cell evolution. This phenomenon, termed immune escape, can compromise the accuracy and reliability of MRD detection by methods like FCM, which depend on identifying surface markers like CD19 on tumor cells. As a result, in the context of targeted therapies, NGS may offer a more trustworthy means of MRD detection (38). Additionally, NGS not only tracks the clonal evolution of specific tumor genes but also monitors novel mutations with potential therapeutic implications arising during treatment. NGS grants more precise evidence for anticipating disease recurrence, presenting a clear advantage over FCM or PCR-based MRD detection methods (26, 54).

IgNGS was first introduced into clinical practice due to its vast applicability. It has been utilized to identify MRD levels in a range of hematological malignancies, including MM, ALL, CLL, MCL, DLBCL, cHL and CTCL (20, 55, 56). Boasting a sensitivity of 10^-6^, IgNGS can discern a single tumor cell among 10^6^ healthy cells, providing more accurate MRD detection even when the tumor burden is minimal. IgNGS has the capability to identify MRD from various types of samples, including PB, BM Formalin fixed paraffin embedded (FFPE) slide, fresh BM, and lymph node FFPE slide (38, 54). Other than IgNGS, Panel-NGS mandates an initial selection of genes linked to certain lymphoma subtypes, which are then organized into a fixed panel. Following specialized library preparation and tailored bioinformatic pipelines for low-input DNA, panel-NGS can effectively identify prevalent single nucleotide variations (SNVs), insertions, deletions, and chromosomal translocations within specimens. By comparing the normal and variant readouts of the target gene produced from deep sequencing, followed by the application of enrichment methods based on amplification or hybrid capture, we can achieve quantitative detection of circulating tumor DNA (26). For IgNGS or Panel-NGS, the contamination of cfDNA with leukocyte genomic DNA can result in false positive results. The low input DNA amounts in the specimen, background error rates of current sequencing technologies, and lack of predetermined gene mutation sites or panel, which are unknown mutations or rare variants outside the detection range of the kit, may lead to false negative results.

CAPP-Seq stands out as the most widely adopted detection technique in panel-NGS, exhibiting particular proficiency for MRD detection in B-cell lymphoma (57–59). Though IgNGS is more susceptible to low ctDNA levels, the sensitivity of CAPP-Seq NGS tends to be compromised by the background error rate encountered during detection (9). To address this, molecular barcoding and digital error suppression techniques have been integrated to curb background errors, consequently amplifying the sensitivity and specificity of MRD identification (60). CAPP-Seq NGS fundamentally employs duplex sequencing as a strategy to trim the background error rate. This process mandates the consistent identification of single nucleotide variations (SNVs) across both complementary DNA strands. Yet, a mere small fraction of the input cfDNA can concurrently recover both strands of the same DNA molecule. Offering a solution, the Stanford University team introduced phased variants (PVs) to mitigate the background error rate. Contrasting with duplex sequencing, PVs involve spotting consistent SNVs solely on a single DNA strand. Phased-Seq NGS, which leverages PVs, boasts superior tumor cell detection precision, most notably in indolent lymphomas such as FL that present scant ctDNA (10, 53).

Panel-NGS techniques have shown greater sensitivity for MRD detection compared to IgNGS. Research by Scherer et al. illuminated the benefits of CAPP-Seq NGS for gene typing and ctDNA detection in DLBCL patients. In comparative evaluations between CAPP-Seq and IgNGS during the treatment and clinical progression of DLBCL patients, the tumor burden changes detected by CAPP Seq were consistent with imaging results. In contrast, IgNGS recorded false negatives during dynamic monitoring (59). Comparing the two prominent panel-NGS methods, CAPP-Seq and PhasED-Seq, the latter seems superior in sensitivity. An investigation utilized both methodologies to measure ctDNA levels in a DLBCL patient receiving first-line treatment. The results indicated that PhasED-Seq identified ctDNA levels that eluded CAPP-Seq. Remarkably, in comparison to CAPP-Seq and PET, PhasED-Seq expedited the ctDNA detection window by 5-10 months. Furthermore, in a cohort study of DLBCL, patients with negative CAPP-Seq results but positive PhasED-Seq readings experienced markedly poorer prognoses than those testing negative on both methods. Boasting a detection limit reaching as low as 0.0004%, PhasED-Seq shows promise (53).

IgNGS and Panel-NGS can detect the mutation of low TF cfDNA by increasing the depth of sequencing of a limited target mutation sites (common cancer drivers or patient-specific panels). Although these state-of-the-art methods provide detection with high accuracy, many patients with radiographically evident disease do not show detectable ctDNA by deep targeted sequencing. Whole Genome Sequencing (WGS) is a way to increase the breadth of sequencing rather than the depth of sequencing. WGS of appropriate sequencing depth can provide high detection probability even at TFs as low as 10−5 (61). However, inappropriate sequencing depth may result in false negative results.

WGS does not target specific mutation sites, but instead primarily integrates changes in amplifications and deletions occurring throughout the genome, namely copy number aberration (CNA) or copy number variation (CNV). CNA/CNV in somatic cells are hallmarks of cancer, with their profiles being predictive of lymphoma outcomes. CNA/CNV can be effectively profiled with Low-pass whole-genome sequencing (LP-WGS) of cfDNA at low coverage. And tumor fraction can be estimated by analyzing CNA/CNV data through bioinformatics pipelines such as ichorCNA. The estimated tumor fraction can be used to measure ctDNA in sample. Up to now, CNA/CNV profiles and tumor fraction estimated from LP-WGS of cfDNA has been utilized in several studies involving HL patients with newly diagnosed, primary mediastinal large B-cell lymphoma (PMBL) and relapsed or refractory large B-cell lymphoma (R/R LBCL) patients with receiving standard-of-care CART19 therapy (62–64).

### Other methods

Time-of-flight mass cytometry (CyTOF), also referred to as mass spectrometry cytometry, blends flow cytometry with mass spectrometry, facilitating a multiparametric analysis of individual cells. By leveraging antibodies tagged with rare or non-biological heavy metal isotopes, CyTOF reduces background noise, sidestepping the challenges posed by overlapping fluorescence signals. Impressively, it simultaneously measures up to 45 parameters, while theoretically holding the capacity to analyze up to 100. Unique to CyTOF, channel crosstalk is absent, and there’s no need for compensation calculations, greatly enhancing the speed of analysis (65). To date, CyTOF’s capabilities have been harnessed to dissect the immunophenotype of tumor cells in conditions like DLBCL, FL, and splenic marginal zone lymphoma (SMZL) (66–70). It holds promise in uncovering tumor heterogeneity, potentially revealing key immunophenotypic and functional transitions during cancer evolution, progression, and metastasis. Thus, CyTOF emerges as a potential tool for lymphoma MRD detection. However, its merits come with constraints. Compared to fluorescence-based flow cytometry, CyTOF’s sensitivity is somewhat compromised, exhibiting a ten-fold dip in performance. Sample and instrument processing can result in significant cell loss, leaving less than half the cells available for evaluation. Moreover, the considerable cost and restricted availability of CyTOF stand as impediments to its broad-scale adoption (65).

Raman spectroscopy is fundamentally based on the interaction between light and the vibrational energy of molecular chemical bonds. Not only does Raman spectroscopy offer an unlabeled, non-invasive, and cost-effective method for the objective detection of MRD and tracking disease progression, but it also sensitively reflects alterations in proteins, DNA, and RNA. However, the traditional Raman signal tends to be faint. To address this, surface-enhanced Raman scattering (SERS) has been introduced. This technique amplifies molecular Raman signals using metal nanostructures. SERS has emerged as a promising technique in aiding the diagnosis and staging of diverse cancers, encompassing prostate, ovarian, breast, lung, and various hematological malignancies such as APL, CLL, and DLBCL (71–73).In a notable study, SERS were utilized to record the Raman spectra from serum samples of healthy controls and DLBCL patients across different disease progression stages. Distinct variances were observed in the spectral intensities of Raman peaks between the two cohorts. Moreover, these intensities demonstrated a strong correlation with the stages of disease progression (74). With the development of Raman spectroscopy, SERS promises to be a valuable instrument for monitoring prognosis and holds particular promise for tracking MRD in lymphoma patients. It possesses the potential to discern between patients with malignant tumors and healthy subjects. Yet, there’s a noticeable gap in research exploring SERS’s capability as a tool to detect MRD during therapy.

**Tables 1**, **2** present a comparison of several prevalent MRD detection methods, highlighting their respective advantages and disadvantages. MFC and RQ-PCR are used for MRD assessment by assaying the CTCs, while ddPCR and NGS-based methods are used for MRD assessment by assaying the ctDNA. The levels of ctDNA are lower in indolent B-cell lymphomas compared to levels in aggressive lymphomas (21, 47). Hence, the enhanced sensitivity of MRD detection is pivotal for tailored and precise treatments under MRD guidance. For indolent lymphoma, MRD detecting attributes to assess treatment response during treatment. For example, patients with MRD-negative are likely in molecular remission. Such outcomes could lead to reductions in drug dosages or shorter chemotherapy sessions, ultimately minimizing side effects and healthcare expenses. On the contrary, MRD-positive patients necessitate additional interventions, including consolidation therapy or immunotherapy. Although there is currently not enough clinical evidence to confirm the effect of MRD assessment in escalating or downgrading treatment, this is the direction of future research. Besides, MRD dynamic monitoring is conducive to identifying molecular recurrence. It identified the risk of recurrence at a median of 3.5 months (range 0-200) before evidence of clinical disease (55). For aggressive lymphoma, in addition to the above, especially in relapsed and refractory lymphomas, dynamic MRD detecting redound to identify clonal evolution and explore the mechanism of drug resistance.

**Table 1:** Comparison of MRD detection methods in some lymphoma subtypes.

	HL	DLBCL	FL	MCL
**Image methods**	Yes	Yes	Yes	Yes
	PET-CT scan or CT scan contrast	PET-CT scan or CT scan contrast	PET-CT scan or CT scan contrast	PET-CT scan or CT scan contrast
**Histologic/pathologic methods**	No, unless there is BM involvement at diagnosis	BM biopsy (optional)	BM biopsy (optional)	BM biopsy (optional)
**MFCM methods**	No	No	No	Yes -The sensitivity is less than RQPCR
**PCR-based methods**	No Advanced HL may be detected	Yes RQPCR ddPCR	Yes ddPCR	Yes RQPCR ddPCR
**NGS methods**	NGS liquid biopsy, investigational use	NGS liquid biopsy, investigational use	NGS liquid biopsy, investigational use -relatively low ctDNA concentrations as compared with other lymphoma subtypes	NGS liquid biopsy, investigational use
**Low depth WGS method**	investigational use	investigational use	investigational use	investigational use
**References**	(8, 75, 76)			

HL, Hodgkin lymphoma; DLBCL, Diffuse large B-cell lymphoma; FL, Follicular lymphoma; MCL, Mantle cell lymphoma; MFCM, Multicolor flow cytometry; NGS Next generation sequencing; WGS, Whole-genome sequencing; PET-CT, Positron Emission Tomography-computed Tomography; CT, Computed Tomography; BM, Bone marrow; RQPCR, Real-time quantitative polymerase chain reaction; ddPCR, Digital droplet polymerase chain reaction; ctDNA, Circulating tumor DNA.

**Table 2:** Comparison of advantages and disadvantages of MRD detection methods.

Methods	FCM-based	PCR-based		NGS-based		
**Techniques**	MFCM	ASO-RQ-PCR	ddPCR	IgNGS	Panel NGS	LP-WGS
**Approximate LOD**	^4-^color:10^-3^ 8-color:10^-4^	10^-4^~10^-5^	10^-5^	10^-6^	10^-6^	10^-3^~10^-5^
**Evaluable lesions**	Immunophenotype	SNVs Translocations		SNVs、Indels、Translocations Copy number variants		Copy number variants Fragmentation profile
**Clonal evolution**	Cannot be tracked	Cannot be tracked	Limited by throughout	Can be tracked		Cannot be tracked
**Cost**	Relatively cheap	Relatively cheap	Relatively expensive	Expensive		
**Advantages**	-Rapid quantification -availability	-Low calibration failure -Accurate -Relatively high sensitivity and reproducibility -Fewer technical requirements	-Rapid -Relatively high sensitivity -Absolute quantification	-High sensitivity -Can detect MRD not identified by MFC or RQ-PCR -Patients specific primers unnecessary -Commercial availability -Analysis is Objective and automatically completed by the software	-High sensitivity -Applicable to all lymphoma subtypes	-Relatively high sensitivity -Relatively rapid -Relatively low cost
**Disadvantages**	-Comparative and insensitive -Lack of standardization -Require presence of CTCs -High professionalism	-Primer design necessary -Relative quantification -Assessment is limited to few genetic lesions	-Requires specialized technology not widely accessible -Relatively complicated steps -Limited capability for multiplexing -Application range depends on the panel selected	-Relatively complicated steps -Requires tumor tissue for clonotype determination	-Research laboratories available -Sensitivity limited by breadth of genes and sequencing depth -Limited detection at low allele frequencies	-Sensitivity limited by sequencing depth -Assessment is limited by the DNA input

MFCM, Multicolor flow cytometry; ASO-RQ-PCR, Allele-specific oligonucleotide real-time quantitative polymerase chain reaction; ddPCR, Digital droplet polymerase chain reaction; IgNGS, immunoglobulin next generation sequencing; Panel NGS, panel-directed next generation sequencing; CAPP-Seq, Cancer personalized profiling by deep sequencing; Phased-Seq, Phased variant enrichment and detection sequencing; LP-WGS, Low-pass whole-genome sequencing; LOD, Limit of detection; SNV, Single nucleotide variation; CTC, Circulating tumor cell.

## MRD analysis

### Analysis of MRD by MFC

The processed samples are stained with a pre-specified panel of fluorescent antibodies related to lymphoma subtypes. Serial dilution experiments of samples can define the MFC panel’s sensitivity for MRD detection (37, 77). Positive MRD was defined as a homogeneous cluster of ≥20 cells with the immunophenotypic characteristics defined at diagnosis. MRD was quantified by dividing the number of lymphoma cells by the total number of events acquired (37, 41).

### Analysis of MRD by RQ-PCR

The diagnostic samples are screened initially by consensus PCR to detect chromosome translocation and/or clonal immunoglobulin rearrangement suitable for MRD assessment. In patients with a detectable clonal marker, sequencing of clonal immunoglobulin rearrangements is done for the design of allele-specific oligonucleotides (ASO) for RQ-PCR. All assays are usually designed with a sensitivity of ≤10^−5^, but are accepted for analysis when a sensitivity of ≤10^−4^ can be reached (78, 79). For determining the quantitative MRD levels, target copy numbers were related to a calibration curve consisting of serial dilutions of the diagnostic sample with known target copy numbers for each individual patient (80). Using a 1 Ct cut-off from background usually was adopted for protocols that aim at therapy reduction. Samples which were positive but not quantifiable were defined as BQR (below quantitative range) (41, 44, 81).

### Analysis of MRD by ddPCR

The methods of extracting DNA are similar to NGS-based. Cell-free DNA was first quantified. A minimum amount of 25 ng of cell-free DNA was required for ddPCR. The adapters contained a sample-specific barcode, and a unique molecular identifier (UMI) is then added to the DNA fragment. Amplification and sequencing of patient-specific V(D)J-regions of the immunoglobulin heavy chain as well as generation of consensus sequences (82, 83). The cell-free DNA was analyzed in triplicate, including positive controls, negative controls, and non-template controls. Samples were considered positive only when a fluorescence signal in the FAM channel was similar to the FAM signal in the positive control and the negative controls confirmed no signal (1, 41, 83, 84). The ddPCR data collected was subjected to bioinformatic analyses. A single threshold to discriminate between positive and negative droplets was established manually for all the samples in the analysis and set above the background signal (85). Variant allele frequency (VAF) of each target in ddPCR reactions was quantified by dividing the merged concentration (copies/20µl) of target by the sum of merged concentration of target plus the merged concentration of the corresponding wild-type assay. The false positive rate (FPR) for each assay was designated as the VAF of target detected in normal cfDNA wells. Patient cfDNA samples were called positive if following criteria were fulfilled: the VAF of the target analyzed was above the FPR for the target assay; the 95% CI Poisson error bars of concentration were non-overlapping between normal plasma and patient cfDNA sample; and at least three positive droplets were observed in total with at least 1 single positive droplet (86).

### Analysis of MRD by NGS

The appropriate kit is selected to extract DNA from processed samples. For example, DNA was extracted from PB/CSF samples using the QIAamp Circulating Nucleic-Acid Kit (Qiagen, Hilden, Germany). DNA was extracted from cell pellet using the QIAamp DNA Blood Mini Kit (Qiagen) or QIAamp DNA Mini Kit (Qiagen) or Qiagen DNeasy Blood and Tissue kit. DNA was extracted from tumor tissues using QIAmp DNA FFPE Advanced UNG Kit (Qiagen) or AllPrep DNA/RNA FFPE Kit (Qiagen) or AllPrep DNA/RNA Mini Kit (Qiagen) (11, 19). Then, individual library preparations, hybridizations and captures were performed for each sample. Concentrations of ctDNA were calculated by multiplying the mean mutated allele frequency (mAF) per patient with the concentration of cfDNA in pg/mL. The result of this calculation was then divided by 3.3 based on the weight of one haploid human genome (3.3pg) to obtain the ctDNA concentration expressed in haploid genome equivalents (hGE) per mL plasma. For visualization purposes, results were transformed into log10 values (11, 19). MRD was often defined to be negative if a negative change of 2 or more log10 levels from baseline was achieved (19).

## Clinical application

**Figure 2:**
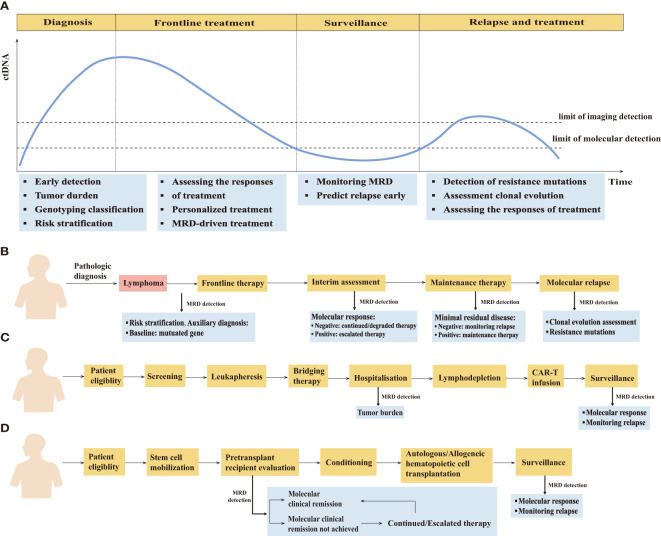
The clinical application of minimal residual disease detection in the management of lymphoma patients. **(A)** Schematic of the clinical time course of representative lymphoma patients and potential clinical applications of minimal residual disease detection at different clinical time course. **(B)** MRD-guided clinical management in lymphoma. **(C)** Clinical application of MRD detection in patients undergoing CAR-T therapy. **(D)** Clinical application of MRD detection in patients undergoing hematopoietic cell transplantation therapy.

MRD detection has a wide range of applications throughout the clinical courses of lymphoma patients (**Figure 2**) (**Supplementary Table S1**).

### MRD prognostic value and risk stratification

The clinical remission rate of lymphoma patients has markedly increased due to the advent of numerous novel therapeutic interventions. Consequently, achieving the highest possible degree of molecular remission has become a common goal among clinicians. Accurate prediction of patient prognosis is essential for individualized treatment, but traditional risk stratification systems have demonstrated limited effectiveness in this regard (59, 87, 88). A genuinely feasible clinical assessment criterion is imperative for achieving a favorable prognosis. A recent study confirmed the prognostic value of MRD in anaplastic lymphoma kinase (ALK)-positive anaplastic large-cell lymphoma (ALCL). It categorizes patients into three groups: minimal disseminated disease (MDD)-negative/MRD-negative, MDD-positive/MRD-negative, and MDD-positive/MRD-positive (89). Early detection of MRD allows clinicians to identify patients at increased risk of recurrence, enabling earlier intervention and ultimately improving patient prognosis.

MRD serves as an independent prognostic biomarker. In one particular study, ctDNA was integrated into the PINK-E prognostic scoring system to create a new prognostic prediction model for ENKTL, termed PINK-EC. When compared with traditional ENKTL prognostic scoring systems like the IPI, the Korean Prognostic Index (KPI), and PINK-E, PINK-EC showed superior prognostic evaluation and clinical application value. This makes PINK-EC a valuable tool in guiding treatment decisions and tailoring personalized therapeutic strategies for ENKTL patients (90). Research assessing the correlation between MRD and PFS and OS in MCL patients revealed that MRD-positive detection after induction and consolidation treatment corresponded to worse PFS and OS outcomes (43, 91). Further investigation validated the prognostic value of MRD in SMZL. The 5-year PFS and 5-year OS in uMRD (undetectable MRD levels by MFCM) patients were notably higher than MRD-positive patients. Moreover, MRD-positive patients achieving partial remission (PR) had a significantly lower 5-year PFS compared to uMRD PR patients. These findings suggest that uMRD may act as an independent prognostic factor in SMZL, especially for those who only reach PR (92). A study on DLBCL found a strong correlation between MRD levels and key clinical factors such as Ann Arbor stage, serum LDH levels, and MTV as measured by PET-CT. Remarkably, MRD was detectable even in samples with normal or low LDH levels, showcasing its superior sensitivity as a biomarker for DLBCL. Elevated pretreatment ctDNA was also significantly linked to shorter PFS in DLBCL patients (59). Additional research on ENKTL confirmed that higher plasma ctDNA levels often signify poorer PFS and OS (90). In conclusion, the potential role of MRD as a prognostic biomarker is clear, but further validation and refinement will help to solidify its role in the clinical management of lymphoma.

Currently, there is significant debate surrounding the management of patients with grade 3a FL. The classification of grade 3a FL as either indolent or aggressive remains uncertain. The baseline level of ctDNA could act as a potential predictor for assessing the invasiveness of lymphoma. Evidence shows that ctDNA levels in FL are typically lower compared to other, more aggressive B-cell lymphomas. This comparison is made by analyzing diagnostic tissue samples with pretreatment plasma samples. Additionally, patients with a favorable prognosis generally exhibit lower levels of plasma ctDNA than those with an unfavorable prognosis. Specifically, a higher detection rate of ctDNA is observed in patients who experience early disease progression within 24 months of diagnosis or those who have not yet achieved complete remission. The reduced ctDNA levels noticed before treatment might indicate the smaller tumor volume and less aggressive characteristics of this lymphoma subtype (10). The treatment of grade 3a FL patients should be personalized. Further research is needed to investigate whether pre-treatment ctDNA levels could serve as a biomarker and aid in treatment decision-making for grade 3a FL patients.

### Dynamic monitoring

MRD is a promising prognostic biomarker that complements traditional clinical indicators. Unlike the single predictor of pretreatment, dynamic monitoring of MRD enables the assessment of disease remission levels, the selection of the most beneficial therapeutic regimens, timely adjustments based on disease response, monitoring of disease recurrence, and participation in the design of clinical research.

Dynamic tracing of ctDNA levels can be employed to monitor disease response. A prospective clinical study has demonstrated that the baseline ctDNA level can function as an independent predictive biomarker or be integrated with the IPI score. Particularly, dynamic monitoring of ctDNA changes in FL patients may provide insights into treatment response and the potential for early progression or relapse (10). The study further explored the correlation between post-treatment ctDNA levels and disease progression or recurrence in 94 PTCL patients undergoing continuous plasma ctDNA monitoring. The findings revealed a significant association between these factors. Notably, patients with a post-treatment ctDNA level reduction of ≥1.5log experienced better survival outcomes compared to those whose levels decreased by ≤1.5log relative to their baseline status (15). Besides, the role of ctDNA MRD in the response assessment has been studied in diverse lymphoma subtypes such as cHL, DLBCL, MCL, CLL/SLL, MF/SS (2, 57, 79, 93–98) (**Supplementary Table S1**). Future strategies for leveraging ctDNA MRD for treatment escalation or de-escalation are likely to be efficient.

At present, 18F-FDG PET-CT has become the primary method for assessing treatment response in most lymphoma patients according to the Lugano Classification, providing insights into tumor size, location, and patient prognosis (99, 100). While patients with complete metabolic response (CMR) may still face relapse, long-term remission can be observed in some PET-positive patients (56). This inconsistency reveals a pressing need for novel biomarkers that can more effectively discern patients most likely to benefit from ASCT and post-ASCT maintenance therapy. Several studies have demonstrated its potential, particularly highlighting the efficacy of rituximab in eliminating MRD and enhancing survival rates for patients undergoing post-ASCT consolidation therapy (43). Post-ASCT MRD monitoring offers a valuable tool to identify patients with a poor prognosis who could benefit from further consolidation therapy. At the same time, it aids in preventing unnecessary toxic effects in patients who have achieved a CR after ASCT alone (43, 56). Moreover, MRD status during the initial year following ASCT carries significant prognostic implications on subsequent PFS outcomes. Impressively, the prognostic influence of MRD status post-ASCT even surpasses that of pre-ASCT and stands independent of the MIPI score and clinical CR (101). Response assessment 1 month after CAR T-cell therapy is critical to guide the clinical treatment strategies. However, interpretation of 18F-FDG PET/CT scan imaging can be challenging because of the lack of specificity of FDG-avid lesions, which may represent tumor, infection, and/or inflammation. In a retrospective study, Erin A et al. (102) examine the relationship between MTV and ctDNA before and after axicabtagene ciloleucel (axi-cel) in patients with R/R LBCL. The lack of correlation between ctDNA and MTV at 1 month, but not 3 months, may be contributed to localized inflammation caused by the CAR T-cell therapy clearing tumor for those patients with either persistent MTV, ctDNA, or both. This study showed that plasma ctDNA might serve as a valuable supplementary test to standard 18F-FDG PET/CT scan imaging at 1 month.

MRD can be utilized to monitor disease relapse in lymphoma patients. In one prospective clinical trial involving DLBCL, IgNGS was used to detect MRD among patients who achieved CR following first-line treatment. The study revealed that patients remaining MRD-negative after CR had a reduced likelihood of disease relapse. Moreover, a sustained MRD-negative status in PB samples was a strong predictor of continued CR for the following 12 months, boasting a negative predictive value for relapse of 97% (20). In another study, Taranto et al. (56) established that MRD is a specific predictor of impending relapse in cHL. They found that all MRD-positive patients eventually relapsed, and MRD levels were significantly associated with quantified tumor burden, as evidenced by PET measurements in a patient with radiation-measurable disease. Further evidence comes from an analysis of ctDNA in a cohort of 20 lymphoma patients using NGS. The study uncovered that ctDNA positive group patients had a notably higher incidence of relapse/PD (7/12, 58.3%) and a lower CR/PR (1/12, 8.3%) compared to the negative group (0, 0%) (5/8, 62.5%) (16). Additionally, the continuous surveillance of ctDNA in patients’ plasma after completing induction therapy indicated that molecular relapse occurred on average 3.5 months prior to clinical relapse (2, 55). The utility of MRD in relapse monitoring has been studied in diverse lymphoma subtypes such as FL, PTCL, T cell lymphoma, MCL (103–106) (**Supplementary Table S1**).

MRD can serve as a surrogate endpoint in clinical trials to guide new treatment strategies or risk-adapted treatment strategies, focusing on molecular remission to investigate the efficacy of innovative treatment regimens. For instance, Torka et al. (3) used MRD negativity as a secondary endpoint in a clinical trial for newly diagnosed mantle cell lymphoma patients. They were treated with a combination of ofatumumab with Hyper-CVAD (hyper-fractionated cyclophosphamide, doxorubicin, vincristine, and dexamethasone) or HD-MA (high dose methotrexate and cytarabine), and high response rates (ORR, 86%; CR, 73%) were achieved, along with MRD negativity rates of 96% at the end of induction therapy. In another study, Soumerai et al. (107) utilized MRD as the primary endpoint to evaluate the efficacy of the BOVen regimen (venetoclax, obinutuzumab, and bendamustine) as initial treatment for untreated CLL/SLL. They also explored the guiding role of MRD during treatment. Their study supports further evaluation of the BOVen regimen in CLL/SLL with treatment duration guided by early MRD response kinetics. 

Moreover, MRD may serve as a biomarker to guide the treatment duration. Giné Eva et al. (108) evaluated the response of ibrutinib combined with rituximab (IR) in the upfront treatment of MCL. They try to use a MRD-driven approach to limit treatment duration. Ibrutinib could be discontinued after 2 years in the case of sustained undetectable MRD. And the study showed that frontline IR achieves a high rate of CRs and undetectable MRD in MCL. Discontinuation seems appropriate in cases with undetectable MRD, except for TP53-mutated cases. Similarly, Matthew S et al. (109) evaluated the response of acalabrutinib, venetoclax, and obinutuzumab in the frontline treatment of CLL/SLL by means of MRD-driven treatment strategy. Treatment could be discontinued at day 1 of cycle 16 or day 1 of cycle 25 if the patient had undetectable MRD in the BM. There are other similar prospective studies to authenticate the effect of MRD-driven treatment (107, 110–113).

## Future directions and conclusions

With the advent of precision medicine, the advancement of new technologies has enhanced the sensitivity and specificity of MRD detection, rendering it a crucial component in the overall treatment strategy for lymphoma. Although different methods have specific indications, standardized techniques are vital across all methods to ensure the reproducibility of MRD detection results. Since MRD detection cannot rely on a single marker, especially for patients lacking immune or molecular markers, the combined application of various detection technologies is essential, leading to a more substantial clinical significance by maximizing the use of information. Among the current methods, NGS-based lymphoma MRD detection stands out for its extensive applicability, leveraging the common characteristics of lymphoid malignancies. Despite its advantages, it still confronts challenges such as complex processes, multiple influencing factors, high costs, and the absence of unified detection and quality control standards. Consequently, it cannot yet supplant imaging examinations, and the widespread clinical implementation may require more time.

In conclusion, MRD is a potential tool in the management of lymphoma. It aids in auxiliary diagnosis, pre-treatment prognosis prediction and risk stratification, on-treatment disease response assessment, post-treatment recurrence monitoring, and the design of clinical trials. This multifaceted utility of MRD facilitates individualized care of lymphoma. An increasing number of studies have explored the value of MRD-driven therapy in MCL, CLL/SLL, ALL, MM, and tried to solve the problems of when to stop treatment, escalation and de-escalation of treatment. But the journey toward making MRD monitoring a clinical tool for guiding treatment is still ongoing. More prospective clinical trials are required to verify the effectiveness of early intervention therapy for lymphoma patients who have experienced molecular relapse but not yet clinical relapse, as well as to assess the potential for unnecessary toxic reactions. The attempt to use MRD to guide the selection and adjustment of treatment plans must be tested in additional prospective clinical trials. Taking into account the relationship between MRD levels and tumor burden, in-depth research on appropriate sampling time points is necessary to optimize MRD monitoring in future trials. As further research progresses, the potential of MRD monitoring for clinical applications can be better validated, laying a robust foundation for its comprehensive integration into clinical practice.
