# Supplementary material for: Minimal residual disease detection in lymphoma: methods, procedures and clinical significance
Source: Front Immunol. 2024 Aug 12;15:1430070. doi: 10.3389/fimmu.2024.1430070 (PMC11345172; doi:10.3389/fimmu.2024.1430070)
Supplement: Supplementary file 1 [file Table1.doc]

**Supplemental Table:**

**Table S1: Selected studies in the MRD monitoring of lymphoma**

| **Author** | **subtype** | **Total patient count** | **technique** | **Monitoring time points** | **intervention** | **analyte** | **Reported data on MRD** | **Clinical applications** |
| --- | --- | --- | --- | --- | --- | --- | --- | --- |
| Lynch et al. (1) | cHL | 30 | PhasED-seq CAPP-Seq FDG-PET/CT | baseline, after C1 and C2, EOT | APVD | PB (plasma) | MRD negativity(C3D1→EOT): Overall: 81%(21/26)→92%(24/26) early-stage favorable disease: 88% (7/8)→100% (8/8) early unfavorable/advanced stage disease: 78% (14/18)→ 89%(16/18) | tumor burden; MRD monitoring during and after therapy |
| Cherng HJ, et al. (2) | DLBCL | 53 | PhasED-seq CAPP-Seq | baseline,on-treatment | LO-CHOP | PB (plasma) | baseline ctDNA detected rate: 94% (31/33)  MRD negativity after at least 5 cycles of therapy: 89% (16/18) | tumor burden; response assessment; molecular subtypes |
| Moreno C, et al. (3) | CLL/SLL | 129 | FCM | on-treatment, post-treatment | IO/CO | PB and/or BM | MRD negativity is one of the secondary endpoints. MRD negativity (IO vs. CO): Overall: 38%（43/113）vs. 25%（29/116） PB: 33%（37/113）vs. 20%（23/116） BM: 25%（28/113）vs. 17%（20/116） CR: 60%（28/47）vs.75%（15/20） PR: 25%（14/56） vs. 19%（14/74） High risk population: 65% (15/23) vs. 27% (3/11) | clinical trial endpoint; Response assessment |
| Lakhotia, et al. (4) | MCL | 53 | ASO-qPCR NGS FDG-PET/CT | pretreatment, after the bortezomib window, after C1 and C2 of induction therapy, EOI, and at each follow-up visit. | bortezomib, DA-EPOCH, rituximab | PB(serum), FFPE, BM clot, frozen tumor cells | pretreatment samples clonotype identified rate: 96% (50/52) | tumor burden; prognosis prediction; ctDNA dynamics |
| Torka, et al. (5) | MCL | 37 | HS-FCM PET/CT | baseline, after C2,4, EOT/before stem cell collection, on day 100, every 6 months for 3 additional years or until disease progression | O-Hyper CVAD, HDMA, HDC-ASCT | PB and/or BM | MRD negativity is one of the secondary endpoints. HSFCM-CR rate at EOI: 71% (20/28) Overall MRD negativity rate: after 2 cycles:78%→post-induction:96%→post-ASCT:89% | prognosis prediction; clinical trial endpoint |
| Giné et al. (6) | MCL | 50 | ASO-qPCR NGS | Every 6 months for a maximum planned follow-up of 7 years. | IR | PB BM | MRD negativity is one of the secondary objectives. MRD negativity: -PB:93%(37/40) - BM:70%(26/37) | MRD–directed treatment; clinical trial endpoint; |
| Siddiqi et al. (7) | CLL/SLL | 25 | FCM NGS | at day30 and month3, 6,9,12,15,18,24 | Lisocabtagene maraleucel | PB BM | MRD negativity:  PB:75% (15/20)  BM: 65% (13/20) | response assessment |
| Herrera et al. (8) | DLBCL | 33 | CAPP-Seq PET/CT | baseline, EOT | pola-BR or BR | PB (plasma) | \ | risk stratification; relapse prediction |
| Luminari S, et al. (9) | FL | 807 | nested PCR FDG-PET/CT | EOT, month6,12,18,24,36 | R-CHOP/BR | PB BM | MRD negativity is prognostic factor for PFS. | prognosis and relapse prediction |
| Soumerai JD, et al. (10) | CLL/SLL | 39 | 14-colour FCM clonoSEQ | PB:C3D1, every other cycle ,EOT,every 3 months during surveillance BM:C3D1, every other cycle,at PB MRD negativity | BOVen | PB BM | MRD negativity is primary endpoint. MRD negativity:  89% (33/37) | MRD–directed treatment |
| Miljkovic MD, et al. (11) | PTCL | 54 | NGS | baseline, EOT, before every cycle, EOI, every3-4/6-12 months during surveillance | DA-EPOCH | PB (serum), FFPE | ctDNA to predict treatment failure after 2 cycles: sensitivity: 75%; specificity:50%; PPV:60%; NPV:67%  ctDNA to predict treatment failure at EOT:  sensitivity: 59%; specificity:86%; PPV:89%; NPV:52% | relapse monitoring |
| Davids MS, et al. (12) | CLL/SLL | 37 | 4-color FCM | baseline, C4D1, C8D1, C16D1, C25D1, every3 months during surveillance | acalabrutini, venetoclax, and obinutuzumab | PB BM | MRD negativity: C8D1: 14% (14/37) →C16D1:38% (14/37) → C25D1:38% (14/37) | MRD–directed treatment; clinical trial endpoint; response assessment |
| Zhang W, et al. (13) | T cell lymphoma | 30 | NGS | on-treatment, post-treatment | CHOPE, chidamide | PB BM | \ | response assessment; relapse monitoring |
| Spiegel JY, et al. (14) | LBCL | 38 | FCM | pretreatment, d0, 7, 14, 21, 56, 90 | CD19-22.BB.z-CAR | PB (plasma) | MRD negativity:  B-ALL: 88% ; LBCL: 29% | response assessment |
| Genuardi E, et al. (15) | MCL FL | \ | MFCM | baseline, during clinical follow-up | \ | PB BM | Baseline clonotype identified rate:  BM: 80%(16/20); PB : 40%(8/20) | clonality assessment; MRD monitoring |
| Genuardi E, et al. (16) | MCL | 20 | IgNGS | \ | Lenalidomide | PB BM | \ | clonality assessment; MRD monitoring |
| Le Gouill S, et al. (17) | MCL | 48 | ASO-qPCR | after C3 and C6 | venetoclax, ibrutinib and obinutuzumab | PB BM | MRD negativity:  after C3: 81%(26/32) | response assessment |
| Le Gouill S, et al. (18) | MCL | 86 | qPCR | EOI | obinutuzumab plus DHAP | BM | at EOI:  MRD negativity: 75%(55/73); MRD-positive:25%(18/73) MRD negativity is primary endpoint. | response assessment; clinical trial endpoint |
| Weng WK, et al. (19) | MF  SS | 35 | NGS | baseline, posttransplant at day +30, +60, +90, +180, +270, +360, and then yearly. | TSEBT-TLI-ATG, allogeneic transplant | PB  skin samples | MRD negativity: 43% (13/30) | response assessment |
| Frank MJ, et al. (20) | MCL | 65 | clonoSEQ | every 3 months in the first year after ASCT and every 6 months thereafter for an additional 2 years or until evidence of clinical PD. | irradiated, CpG-activated tumor cells, ASCT | PB | 1 year after ASCT: MRD negative:89% MRD negativity is primary endpoint. | response assessment; clinical trial endpoint |
| Davids MS, et al. (21) | CLL/SLL | 85 | MFCM | after C3, 2 months after the final cycle, every 6 months during surveillance. | Ibrutinib, FCR | PB BM | MRD negativity is primary endpoint. MRD-negative: 2 months after the last cycle: 33% (ibrutinib plus FCR) VS. 20% (FCR alone)  Overall: 84% (71/85) in BM | MRD–directed treatment; clinical trial endpoint; response assessment |
| Roberts AW, et al. (22) | CLL/SLL | 436 | MFCM | on-treatment, post-treatment | venetoclax | PB BM | MRD negativity: 27% in PB; 16% in BM | response assessment; prognosis prediction |
| Grommes C, et al. (23) | CNS lymphoma | 15 | NGS | baseline, C3D1, C5D1 | ibrutinib, HD-MTX, rituximab | CSF | Sustained tumor responses were associated with clearance of ctDNA from the CSF. | response assessment |
| Gressin R, et al. (24) | MCL | 76 | qPCR | baseline, after C4 and C6, every 3-6 months during surveillance. | rituximab, bendamustine, bortezomib and dexamethasone | PB BM | MRD negativity:（PB vs. BM） C4: 88% (50/57) vs. 77%（37/48) C6: 87% (47/54) vs. 76%（35/46) | response assessment; prognosis prediction |
| Klener, P. et al. (25) | MCL | 73 | qPCR | baseline, after C3 and C6 | R-CHOP and R-cytarabine | PB BM | MRD negativity:（PB vs. BM） C3: 49.1% vs. 39.2% C6: 77.8% vs. 54.5% | response assessment |
| Ruan J, et al. (26) | MCL | 38 | clonoSEQ | follow-up | LR | PB | MRD negativity after ≥3 years of treatment: 80% (8/10) | response assessment; relapse monitoring |
| Chen RW, et al. (27) | MCL | 23 | ddPCR | baseline, on day 1 of each cycle | bortezomib, rituximab | PB | the role of quantitative CCND1 mRNA in monitoring MRD. | response assessment; |
| Tam CS, et al. (28) | MCL | 24 | 8-color FCM  ASO-PCR | on-treatment, post-treatment | oral ibrutinib and venetoclax | PB BM | MRD negativity:（FCM vs. ASO-PCR） 67% vs. 38% | response assessment; |
| Rossi D, et al. (29) | DLBCL | 50 | CAPP-Seq | baseline, on day 1 of each cycle, EOT, progression | R-CHOP | PB (plasma) | \ | tracking clonal evolution |
| Kolstad A, et al. (30) | MCL | 183 | nested PCR qPCR | pre-ASCT and at 2 to 3 months, at 6 months, and then every 6 months post-ASCT, until relapse or completion of 5 years of follow-up | ASCT | PB  BM | \ | relapse monitoring; MRD–directed treatment |
| Sarkozy C, et al. (31) | FL | 34 | IgNGS | baseline | NA | tumor  PB (plasma) | tumour clonotype identification: tumor: 85% (29/34)  PB: 74% (25/34) | clonality assessment; prognosis prediction |
| Herrera AF, et al. (32) | HL NHL | 139 | IgNGS | prior to HSCT and at 1, 2, 3, 6, 9, 12, 18 and 24 months after HSCT, or until relapse. | NA | PB (plasma) | sensitivity/specificity: (at 1,2,3 months after HSCT) 69%/45%→73%/55%→73%/55% ctDNA-positive:(at 1,2,3,6,12,24 months after HSCT) 60%→55%→52%→36%→21%→10% 84%(16/19) was detected ctDNA prior to clinical relapse or progression. | prognosis prediction; relapse monitoring |
| Assouline SE, et al. (33) | DLBCL | 40 | CAPP-seq ddPCR | on-treatment | panobinostat with or without rituximab | PB (plasma) | \ | response assessment |
| Armand P, et al. (34) | MCL | 23 | ClonoSEQ | baseline, after C3 and C6 | RB/RC | PB | \ | response assessment |
| cHL: classical Hodgkin lymphoma; C1:cycle1; C2:cycle2; EOT: end of treatment; APVD: pembrolizumab, doxorubicin, vinblastine, dacarbazine; C3D1:first day in cycle 3; DLBCL: Diffuse large B-cell lymphoma; LO-CHOP: obinutuzumab, lenalidomide, cyclophosphamide, doxorubicin, vincristine, prednisone; ctDNA: Circulating tumour DNA; CLL/SLL: Chronic lymphocytic leukemia/small lymphocytic lymphoma; IO: ibrutinib plus obinutuzumab; CO: chlorambucil plus obinutuzumab; PB: Peripheral blood; BM: Bone marrow; MCL: Mantle cell lymphoma; EOI: end of induction; DA-EPOCH: dose-adjusted etoposide, doxorubicin, cyclophosphamide, prednisone, vincristine; FFPE: formalin-fixed paraffin-embedded; HS-FCM: high-sensitivity flow cytometry assay; O-HyperCVAD: ofatumumab, hyper-fractionated cyclophosphamide, doxorubicin, vincristine, and dexamethasone; HDMA: high dose methotrexate and cytarabine; HDC-ASCT: high dose chemotherapy and autologous stem cell transplant; HSFCM-CR rate: meeting all CR criteria and MRD negative; ASO-qPCR: allele-specific oligonucleotide real-time quantitative polymerase chain reaction; IR: ibrutinib, rituximab; pola-BR: polatuzumab vedotin, bendamustine, rituximab; BR: bendamustine, rituximab; FL: Follicular lymphoma; R-CHOP: rituximab, cyclophosphamide, doxorubicin, vincristine, and prednisone; BR: bendamustine, rituximab; BOVen: zanubrutinib, obinutuzumab, venetoclax; PPV: positive predictive value; NPV: negative predictive value; C4D1: first day in cycle 4; C8D1:first day in cycle 8; C16D1:first day in cycle 16; C25D1:first day in cycle 25; CHOPE: cyclophosphamide, doxorubicin, vincristine, prednisone, and etoposide; LBCL: large B-cell lymphoma; CD19-22.BB.z-CAR: bispecific CAR targeting CD19 and/or CD22; C6: cycle 6; DHAP: dexamethasone, high-dose cytarabine, and cisplatin; TSEBT-TLI-ATG: total skin electron beam therapy, total lymphoid irradiation, and antithymocyte globulin; MF: mycosis fungoides; SS: Sézary syndrome; FCR: Fludarabine, cyclophosphamide, and rituximab; C3D1:first day in cycle 3; C5D1:first day in cycle 5; HD-MTX: high-dose methotrexate; CSF: cerebrospinal fluid; ASCT: Autologous stem cell transplantation; LR: lenalidomide plus rituximab; CCND1:cyclin D1; RB/RC: rituximab/bendamustine or rituximab/high-dose cytarabine; | | | | | | | | |

References:

1. Lynch RC, Ujjani CS, Poh C, Warren EH, Smith SD, Shadman M, et al. Concurrent pembrolizumab with AVD for untreated classic Hodgkin lymphoma. Blood. 2023 May 25;141(21):2576–86.

2. Cherng HJJ, Alig SK, Oki Y, Nastoupil LJ, Fayad L, Neelapu SS, et al. A phase 1/2 study of lenalidomide and obinutuzumab with CHOP for newly diagnosed DLBCL. Blood Adv. 2023 Apr 11;7(7):1137–45.

3. Moreno C, Greil R, Demirkan F, Tedeschi A, Anz B, Larratt L, et al. First-line treatment of chronic lymphocytic leukemia with ibrutinib plus obinutuzumab versus chlorambucil plus obinutuzumab: final analysis of the randomized, phase III iLLUMINATE trial. Haematologica. 2022 Sep 1;107(9):2108–20.

4. Lakhotia R, Melani C, Dunleavy K, Pittaluga S, Saba N, Lindenberg L, et al. Circulating tumor DNA predicts therapeutic outcome in mantle cell lymphoma. Blood Adv. 2022 Apr 26;6(8):2667–80.

5. Torka P, Akhtar OS, Reddy NM, Baysal BE, Kader A, Groman A, et al. Ofatumumab plus HyperCVAD/HD-MA induction leads to high rates of minimal residual disease negativity in patients with newly diagnosed mantle cell lymphoma: Results of a phase 2 study. Cancer. 2022 Apr 15;128(8):1595–604.

6. Giné E, de la Cruz F, Jiménez Ubieto A, López Jimenez J, Martín García-Sancho A, Terol MJ, et al. Ibrutinib in Combination With Rituximab for Indolent Clinical Forms of Mantle Cell Lymphoma (IMCL-2015): A Multicenter, Open-Label, Single-Arm, Phase II Trial. J Clin Oncol. 2022 Apr 10;40(11):1196–205.

7. Siddiqi T, Soumerai JD, Dorritie KA, Stephens DM, Riedell PA, Arnason J, et al. Phase 1 TRANSCEND CLL 004 study of lisocabtagene maraleucel in patients with relapsed/refractory CLL or SLL. Blood. 2022 Mar 24;139(12):1794–806.

8. Herrera AF, Tracy S, Croft B, Opat S, Ray J, Lovejoy AF, et al. Risk profiling of patients with relapsed/refractory diffuse large B-cell lymphoma by measuring circulating tumor DNA. Blood Adv. 2022 Mar 22;6(6):1651–60.

9. Luminari S, Manni M, Galimberti S, Versari A, Tucci A, Boccomini C, et al. Response-Adapted Postinduction Strategy in Patients With Advanced-Stage Follicular Lymphoma: The FOLL12 Study. J Clin Oncol. 2022 Mar 1;40(7):729–39.

10. Soumerai JD, Mato AR, Dogan A, Seshan VE, Joffe E, Flaherty K, et al. Zanubrutinib, obinutuzumab, and venetoclax with minimal residual disease-driven discontinuation in previously untreated patients with chronic lymphocytic leukaemia or small lymphocytic lymphoma: a multicentre, single-arm, phase 2 trial. Lancet Haematol. 2021 Dec;8(12):e879–90.

11. Miljkovic MD, Melani C, Pittaluga S, Lakhotia R, Lucas N, Jacob A, et al. Next-generation sequencing-based monitoring of circulating tumor DNA reveals clonotypic heterogeneity in untreated PTCL. Blood Adv. 2021 Oct 26;5(20):4198–210.

12. Davids MS, Lampson BL, Tyekucheva S, Wang Z, Lowney JC, Pazienza S, et al. Acalabrutinib, venetoclax, and obinutuzumab as frontline treatment for chronic lymphocytic leukaemia: a single-arm, open-label, phase 2 study. Lancet Oncol. 2021 Oct;22(10):1391–402.

13. Zhang W, Wang W, Han X, Gan Y, Qian L, Zhang Y, et al. Circulating tumor DNA by high-throughput sequencing of T cell receptor monitored treatment response and predicted treatment failure in T cell lymphomas. Int J Lab Hematol. 2021 Oct;43(5):1041–9.

14. Spiegel JY, Patel S, Muffly L, Hossain NM, Oak J, Baird JH, et al. CAR T cells with dual targeting of CD19 and CD22 in adult patients with recurrent or refractory B cell malignancies: a phase 1 trial. Nat Med. 2021 Aug;27(8):1419–31.

15. Genuardi E, Klous P, Mantoan B, Drandi D, Ferrante M, Cavallo F, et al. Targeted locus amplification to detect molecular markers in mantle cell and follicular lymphoma. Hematol Oncol. 2021 Aug;39(3):293–303.

16. Genuardi E, Romano G, Beccuti M, Alessandria B, Mannina D, Califano C, et al. Application of the Euro Clonality next-generation sequencing-based marker screening approach to detect immunoglobulin heavy chain rearrangements in mantle cell lymphoma patients: first data from the Fondazione Italiana Linfomi MCL0208 trial. Br J Haematol. 2021 Jul;194(2):378–81.

17. Le Gouill S, Morschhauser F, Chiron D, Bouabdallah K, Cartron G, Casasnovas O, et al. Ibrutinib, obinutuzumab, and venetoclax in relapsed and untreated patients with mantle cell lymphoma: a phase 1/2 trial. Blood. 2021 Feb 18;137(7):877–87.

18. Le Gouill S, Beldi-Ferchiou A, Alcantara M, Cacheux V, Safar V, Burroni B, et al. Molecular response after obinutuzumab plus high-dose cytarabine induction for transplant-eligible patients with untreated mantle cell lymphoma (LyMa-101): a phase 2 trial of the LYSA group. Lancet Haematol. 2020 Nov;7(11):e798–807.

19. Weng WK, Arai S, Rezvani A, Johnston L, Lowsky R, Miklos D, et al. Nonmyeloablative allogeneic transplantation achieves clinical and molecular remission in cutaneous T-cell lymphoma. Blood Adv. 2020 Sep 22;4(18):4474–82.

20. Frank MJ, Khodadoust MS, Czerwinski DK, Haabeth OAW, Chu MP, Miklos DB, et al. Autologous tumor cell vaccine induces antitumor T cell immune responses in patients with mantle cell lymphoma: A phase I/II trial. J Exp Med. 2020 Sep 7;217(9).

21. Davids MS, Brander DM, Kim HT, Tyekucheva S, Bsat J, Savell A, et al. Ibrutinib plus fludarabine, cyclophosphamide, and rituximab as initial treatment for younger patients with chronic lymphocytic leukaemia: a single-arm, multicentre, phase 2 trial. Lancet Haematol. 2019 Aug;6(8):e419–28.

22. Roberts AW, Ma S, Kipps TJ, Coutre SE, Davids MS, Eichhorst B, et al. Efficacy of venetoclax in relapsed chronic lymphocytic leukemia is influenced by disease and response variables. Blood. 2019 Jul 11;134(2):111–22.

23. Grommes C, Tang SS, Wolfe J, Kaley TJ, Daras M, Pentsova EI, et al. Phase 1b trial of an ibrutinib-based combination therapy in recurrent/refractory CNS lymphoma. Blood. 2019 Jan 31;133(5):436–45.

24. Gressin R, Daguindau N, Tempescul A, Moreau A, Carras S, Tchernonog E, et al. A phase 2 study of rituximab, bendamustine, bortezomib and dexamethasone for first-line treatment of older patients with mantle cell lymphoma. Haematologica. 2019 Jan;104(1):138–46.

25. Klener P, Fronkova E, Kalinova M, Belada D, Forsterova K, Pytlik R, et al. Potential loss of prognostic significance of minimal residual disease assessment after R-CHOP-based induction in elderly patients with mantle cell lymphoma in the era of rituximab maintenance. Hematol Oncol. 2018 Dec;36(5):773–8.

26. Ruan J, Martin P, Christos P, Cerchietti L, Tam W, Shah B, et al. Five-year follow-up of lenalidomide plus rituximab as initial treatment of mantle cell lymphoma. Blood. 2018 Nov 8;132(19):2016–25.

27. Chen RW, Palmer JM, Tomassetti S, Popplewell LL, Alluin J, Chomchan P, et al. Multi-center phase II trial of bortezomib and rituximab maintenance combination therapy in patients with mantle cell lymphoma after consolidative autologous stem cell transplantation. J Hematol Oncol. 2018 Jun 28;11(1):87.

28. Tam CS, Anderson MA, Pott C, Agarwal R, Handunnetti S, Hicks RJ, et al. Ibrutinib plus Venetoclax for the Treatment of Mantle-Cell Lymphoma. N Engl J Med. 2018 Mar 29;378(13):1211–23.

29. Rossi D, Diop F, Spaccarotella E, Monti S, Zanni M, Rasi S, et al. Diffuse large B-cell lymphoma genotyping on the liquid biopsy. Blood. 2017 Apr 6;129(14):1947–57.

30. Kolstad A, Pedersen LB, Eskelund CW, Husby S, Grønbæk K, Jerkeman M, et al. Molecular Monitoring after Autologous Stem Cell Transplantation and Preemptive Rituximab Treatment of Molecular Relapse; Results from the Nordic Mantle Cell Lymphoma Studies (MCL2 and MCL3) with Median Follow-Up of 8.5 Years. Biol Blood Marrow Transplant. 2017 Mar;23(3):428–35.

31. Sarkozy C, Huet S, Carlton VEH, Fabiani B, Delmer A, Jardin F, et al. The prognostic value of clonal heterogeneity and quantitative assessment of plasma circulating clonal IG-VDJ sequences at diagnosis in patients with follicular lymphoma. Oncotarget. 2017 Jan 31;8(5):8765–74.

32. Herrera AF, Kim HT, Kong KA, Faham M, Sun H, Sohani AR, et al. Next-generation sequencing-based detection of circulating tumour DNA After allogeneic stem cell transplantation for lymphoma. Br J Haematol. 2016 Dec;175(5):841–50.

33. Assouline SE, Nielsen TH, Yu S, Alcaide M, Chong L, MacDonald D, et al. Phase 2 study of panobinostat with or without rituximab in relapsed diffuse large B-cell lymphoma. Blood. 2016 Jul 14;128(2):185–94.

34. Armand P, Redd R, Bsat J, Mayuram S, Giardino A, Fisher DC, et al. A phase 2 study of Rituximab-Bendamustine and Rituximab-Cytarabine for transplant-eligible patients with mantle cell lymphoma. Br J Haematol. 2016 Apr;173(1):89–95.
